# HIV-1 Nef interaction influences the ATP-binding site of the Src-family kinase, Hck

**DOI:** 10.1186/1472-6769-12-1

**Published:** 2012-03-15

**Authors:** Teodora Pene-Dumitrescu, Sherry T Shu, Thomas E Wales, John J Alvarado, Haibin Shi, Purushottam Narute, Jamie A Moroco, Joanne I Yeh, John R Engen, Thomas E Smithgall

**Affiliations:** 1 Department of Microbiology and Molecular Genetics, University of Pittsburgh School of Medicine, Pittsburgh, PA 15219, USA; 2 Department of Chemistry and Chemical Biology and Barnett Institute of Chemical & Biological Analysis, Northeastern University, Boston, MA 02115, USA; 3 Department of Structural Biology, University of Pittsburgh School of Medicine, Pittsburgh, PA 15260, USA

## Abstract

**Background:**

Nef is an HIV-1 accessory protein essential for viral replication and AIDS progression. Nef interacts with a multitude of host cell signaling partners, including members of the Src kinase family. Nef preferentially activates Hck, a Src-family kinase (SFK) strongly expressed in macrophages and other HIV target cells, by binding to its regulatory SH3 domain. Recently, we identified a series of kinase inhibitors that preferentially inhibit Hck in the presence of Nef. These compounds also block Nef-dependent HIV replication, validating the Nef-SFK signaling pathway as an antiretroviral drug target. Our findings also suggested that by binding to the Hck SH3 domain, Nef indirectly affects the conformation of the kinase active site to favor inhibitor association.

**Results:**

To test this hypothesis, we engineered a "gatekeeper" mutant of Hck with enhanced sensitivity to the pyrazolopyrimidine tyrosine kinase inhibitor, NaPP1. We also modified the RT loop of the Hck SH3 domain to enhance interaction of the kinase with Nef. This modification stabilized Nef:Hck interaction in solution-based kinase assays, as a way to mimic the more stable association that likely occurs at cellular membranes. Introduction of the modified RT loop rendered Hck remarkably more sensitive to activation by Nef, and led to a significant decrease in the K_m _for ATP as well as enhanced inhibitor potency.

**Conclusions:**

These observations suggest that stable interaction with Nef may induce Src-family kinase active site conformations amenable to selective inhibitor targeting.

## Background

Nef is an HIV-1 accessory protein that facilitates virus infectivity, replication, and immune evasion [1-3]. In non-human primate models of AIDS, high-titer viral replication and development of AIDS-like disease requires an intact *Nef *gene [4]. Long-term non-progressive HIV infection in humans is also associated with Nef-defective HIV isolates in some cases [5,6]. Complementary in vivo studies have shown that directed expression of *Nef *alone to HIV target cells induces an AIDS-like syndrome in transgenic mice [7-9]. Taken together, these studies underscore the importance of HIV-1 Nef in AIDS pathogenesis.

Nef is not known to exhibit any intrinsic enzymatic activity. Instead, Nef interacts with multiple host cell signaling pathways to enhance HIV-1 replication and promote AIDS progression [10]. Previous work from our group has identified members of the Src kinase family as direct Nef effectors [11-15]. This kinase family includes Hck, a Src-family member expressed in macrophages, which are a critical HIV target cell type and viral reservoir. Nef interacts with the Hck SH3 domain, leading to constitutive Hck activation that may contribute to macrophage survival, MHC-I downregulation and M-tropic HIV replication [11,12,14,16-18]. Nef has also been shown to bind and activate the Src-family kinases Lyn and c-Src, which exhibit a broader expression pattern including other HIV target cell types [14]. Thus, Nef-dependent activation of Src family kinases is likely to occur in most HIV-infected cells.

Hck shares a similar domain organization and structural architecture with other members of the Src kinase family [19-21]. Key structural features include an N-terminal unique domain with sites for lipid attachment that drive membrane association, followed by the regulatory SH3 and SH2 domains, an SH2-kinase linker, the kinase domain, and a C-terminal negative regulatory tail. Nef binds to the Hck SH3 domain through a bipartite mechanism revealed in structural analyses of Nef:SH3 complexes [22-25]. Nef:SH3 interaction is dependent in part on a highly conserved PxxPxR motif, which forms a polyproline type II helix typical of most SH3 ligands. In addition, the αA and αB helices of Nef form a hydrophobic pocket that interacts with an Ile residue in the RT loop of the SH3 domain. Nef binding displaces the SH3 domain from its negative regulatory position on the back of the kinase domain, leading to kinase activation. Interestingly, mutation of the Nef PxxPxR motif completely abolished development of the AIDS-like phenotype in Nef-transgenic mice [8]. Furthermore, crossing Nef transgenic mice into a *hck-*null background increased the latency for AIDS-like disease onset and decreased mortality [8]. These data provide strong evidence that Src-family kinase activation by Nef is important for AIDS pathogenesis, and identify this signaling pathway as a target for therapeutic intervention.

Recently, we developed a chemical library screening assay based on Nef-dependent activation of Hck in vitro [15]. Using this assay, we identified a series of diphenylfuropyrimidine (DFP) analogs that preferentially inhibit Hck in the presence of Nef. These compounds also potently blocked HIV-1 replication in a Nef-dependent manner [15], validating inhibitors of Nef-SFK signaling as potential antiretroviral agents. Our observation that DFP-based kinase inhibitors selectively inhibit the Nef:Hck complex suggested that Nef binding to the Hck SH3 domain induces structural changes in the kinase domain that favor inhibitor association. In the present study, we developed a system to test this hypothesis directly using a "gatekeeper" mutant of Hck with engineered sensitivity to the pyrazolopyrimidine analog, NaPP1 [26,27]. This mutation involves substitution of the gatekeeper threonine (Thr338; numbering as per c-Src crystal structure [28]) with a much smaller alanine residue (Hck-TA mutant), providing access for NaPP1 to the hydrophobic cavity adjacent to the ATP binding site. This combination of mutant kinase and NaPP1 results in a high degree of inhibitor selectivity and potency both in vitro and in cell-based assays [27]. Because NaPP1 binds to the Hck-TA active site in a specific location, it serves as a chemical probe for conformational changes that may occur in response to Nef binding. In addition to the gatekeeper mutation, we modified the SH3 domain to enhance interaction with Nef [29,30]. This modification enabled stable association of Hck with Nef in solution-based kinase assays, thus mimicking the stable association that is likely to occur between Hck and Nef at cellular membranes [17]. Use of this modified form of Hck combined with the selective inhibitor enabled us to demonstrate that Nef binding results in changes in the K_m _for ATP as well as inhibitor potency. These observations support the idea that Nef binding induces a unique active conformation of the Hck active site that can be targeted with selective inhibitors.

## Methods

### Recombinant protein expression and purification

Nef-SF2 and Nef-Consensus for kinase assays were expressed in *E. coli *with hexahistidine tags and purified by immobilized metal affinity chromatography (IMAC) as described elsewhere [14]. For SPR experiments, the same two Nef proteins were purified without N-terminal His tags by anion-exchange chromatography (HiPrep Q FF; GE Healthcare). Both the His-tagged and untagged Nef proteins were further purified by gel filtration chromatography on a HiLoad 26/60 Superdex 75 column (GE Healthcare) with 20 mM Tris-HCl, pH 8.3, 100 mM NaCl and 3 mM DTT as mobile phase.

A cDNA clone for mouse Hck (mHck) was modified at its gatekeeper position (Thr338) with alanine, the C-terminal tail was changed to the autoregulatory sequence, Tyr-Glu-Glu-Ile (YEEI), and the N-terminal unique domain was replaced with a hexahistidine tag as described previously for human Hck [27]. The coding sequence for the High Affinity RT loop (HART) was then introduced by replacing the wild-type SH3 RT loop sequence EAIHRE with TSPFPW using site-directed mutagenesis [30]. The presence of all changes was confirmed by DNA sequence analysis of the entire Hck open reading frame. The mHck-T338A-YEEI (mHck-TA) and the mHck-T338A-HART-YEEI (mHck-TA-HART) coding sequences were subcloned into the baculovirus transfer vector pVL1392 (BD Biosciences) and the resulting plasmids used to create high-titer recombinant baculoviruses in Sf9 insect cells using Baculogold DNA and the manufacturer's protocol (BD Biosciences). Recombinant mHck proteins were expressed in Sf9 insect cells and purified using a combination of ion-exchange and IMAC chromatography as described previously for human Hck [27]. Following purification, the mHck proteins were dialyzed against 20 mM Tris-HCl, pH 8.3, containing 100 mM NaCl and 3 mM DTT. The purity and concentration of each recombinant protein were confirmed by SDS-PAGE, densitometry and electrospray mass spectrometry.

The sequences encoding the wild-type hHck SH3 domain, mHck SH3 domain, and mHck SH3-HART mutant were amplified by PCR and subcloned into the bacterial expression vector, pET-14b (mHck SH3s) or pET-21a (hHck SH3). All three SH3 proteins were expressed in *E. coli *BL21(DE3)pLysS in the presence of 0.4 mM IPTG for 4 h at room temperature. Cells were sonicated in lysis buffer (20 mM Tris-HCl, pH 8.3, 10% glycerol, and 5 mM 2-mercaptoethanol), and soluble SH3 proteins were purified from clarified cell lysates using a combination of anion-exchange (HiPrep Q FF; GE Healthcare), IMAC (HiTrap Chelating HP; GE Healthcare), and size-exclusion chromatography (HiLoad 26/60 Superdex 75; GE Healthcare). Purified SH3 proteins were stored in 20 mM Tris-HCl, pH 8.3, 100 mM NaCl and 3 mM DTT. SH3 protein purity and concentration were confirmed by SDS-PAGE, densitometry and mass spectrometry.

### Hydrogen exchange mass spectrometry (HXMS)

HXMS was used to investigate Nef:SH3 interaction as described [31] with the following modifications. Recombinant Hck SH3 and Nef proteins were equilibrated together at 4°C for at least 110 min before the initiation of the labeling reaction. Starting reactions consisted of the SH3 (50 μM) and Nef (117 μM) proteins in 20 mM Tris-HCl, pH 8.3, 100 mM NaCl, and 3 mM DTT. Deuterium labeling was initiated by 15-fold dilution of the binding reaction into D_2_O labeling buffer (20 mM Tris, pD 8.3, 100 mM NaCl, 3 mM DTT). Labeled proteins were injected onto a POROS 20 R2 protein trap and desalted with 0.05% trifluroacetic acid (TFA) at a flow rate of 500 μL/min. The proteins were eluted into the mass spectrometer using a linear 15% to 75% (v/v) acetonitrile gradient over 4 min at 50 μL/min with a Shimadzu HPLC system (LC-20AD). HPLC was performed using protiated solvents which results in the removal of deuterium from the side-chains and the amino/carboxy termini which exchange faster than backbone amide hydrogen atoms [32,33]. Mass spectral analyses were carried out with a Waters LCT-Premier^XE ^mass spectrometer with a standard electrospray source, a capillary voltage of 3.2 kV and a cone voltage of 35 V. The deuterium levels were not corrected for back-exchange [32] and reflect relative changes across the protein samples. The isotope envelopes in bimodal patterns were fit with two Gaussian functions whose widths were estimated from a single binomial isotopic envelope before and after the appearance of the bimodal pattern. The unfolding rates for those SH3 domains that presented evidence of a bimodal isotopic envelope were determined from the slope of pseudo-first-order kinetic plots of the decrease in the relative intensity of the lower mass envelope with time [34-36].

### In vitro kinase assay

Protein-tyrosine kinase assays were performed using the FRET-based Z'-Lyte kinase assay kit and Tyr-2 peptide substrate according to the manufacturer's instructions (Life Technologies) and our previous work [14,15,27,37]. All assays were performed in quadruplicate in low-volume, non-binding 384-well plates (Corning). To determine the K_m _for ATP, time course experiments were run at fixed concentrations of substrate (1 μM) and kinase in the presence of an ATP concentration range from 0 to 500 μM. The K_m _for ATP was then calculated from a plot of ATP concentration against initial reaction velocity followed by non-linear regression analysis (GraphPad Prism). For inhibition experiments, the assay was first optimized to determine the amount of kinase or kinase plus Nef (1:10 molar ratio) necessary to phosphorylate ~50% of the Tyr-2 peptide substrate in the presence of an ATP concentration equal to twice the K_m_. Kinases were pre-incubated with NaPP1 in kinase assay buffer (50 mM Hepes, pH 7.5, 10 mM MgCl_2_, and 1 mM EGTA, 0.01% Brij-35) for 30 min, followed by incubation with ATP and Tyr-2 peptide for 1 h at room temperature. Development reagent was then added to the reaction for an additional 1 h at room temperature, followed by addition of the stop reagent. Fluorescence was assessed at an excitation wavelength of 400 nm; coumarin fluorescence and the fluorescein FRET signal were monitored at 445 and 520 nm, respectively. Reactions run in the absence of ATP served as the 0% phosphorylation control, whereas a stoichiometrically phosphorylated Tyr2 peptide was used as the 100% phosphorylation control. Raw fluorescence values were corrected for background, and reaction endpoints calculated as emission ratios of coumarin fluorescence divided by the fluorescein FRET signal. These ratios were normalized to the ratio obtained with the 100% phosphorylation control peptide. IC_50 _values were calculated using a sigmoidal curve fit using GraphPad Prism. Additional details of the Z'-Lyte assay principle and calculation of percent inhibition has been described elsewhere [15,27].

### Surface plasmon resonance (SPR)

Recombinant Hck SH3 domain proteins were exchanged into HBS-EP buffer (10 mM HEPES, pH 7.4, 150 mM NaCl, 3 mM EDTA, 0.05% v/v P20 surfactant) and concentrated with an Amicon Ultra 15 ml 3 kDa molecular weight cutoff spin concentrator. Primary untagged Nef proteins were exchanged into HBS-EPD buffer (10 mM HEPES, pH 7.4, 150 mM NaCl, 3 mM EDTA, 0.05% v/v P20 surfactant, 1 mM DTT) and concentrated with an Amicon Ultra 15 ml 10 kDa molecular weight cutoff spin concentrator. SPR analysis was performed on a BIAcore 3000 instrument (GE Healthcare) using a four-channel CM5 biosensor chip with the Hck SH3 domains covalently attached as ligand via standard amine coupling chemistry [38,39]. Nef proteins (as analyte) were flowed past the immobilized SH3 proteins on the biosensor chip at a flow rate of 10 μl/min for 5 min using Nef protein concentrations above and below the K_D _(see Table 1). The initial binding reaction was followed by dissociation for 5 min, and the chip surface was regenerated using HBS-EPD buffer at a flow rate of 30 μl/min for 10 min. Binding curves recorded at each analyte concentration were assessed in triplicate, corrected for buffer effects, and fit to a 1:1 Langmuir binding model using the BIAevaluation 4.1 software suite (GE Healthcare).

**Table 1:** Evaluation of Nef binding to wild-type and HART SH3 domains by surface plasmon resonance (SPR).

SH3 domain	k_a _(M^-1^s^-1^) × 10^5^	k_d _(s^-1^) × 10^-2^	K_D _(μM)	Nef concentrations (μM)
**Nef-SF2**				

Human Hck SH3	1.60 ± 0.06	1.18 ± 0.04	0.07 ± 0.01	0, 0.007, 0.02, 0.062, 0.19, 0.56, 1.67, 5.0
Mouse Hck SH3	5.54 ± 1.77	7.00 ± 0.36	0.13 ± 0.03	0, 0.007, 0.02, 0.062, 0.19, 0.56, 1.67
Mouse Hck HART	14.20 ± 1.40	1.43 ± 0.01	0.01 ± 0.00	0, 0.002, 0.007, 0.02, 0.062, 0.19

Nef-Consensus*				

Human Hck SH3	10.00 ± 0.80	11.80 ± 0.20	0.12 ± 0.01	0, 0.02, 0.062, 0.19, 0.56, 1.67, 5.0
Mouse Hck SH3	2.41 ± 0.05	1.85 ± 0.11	0.08 ± 0.01	0, 0.007, 0.02, 0.062, 0.19, 0.56, 1.67
Mouse Hck HART	15.70 ± 0.20	3.24 ± 0.16	0.02 ± 0.00	0, 0.002, 0.007, 0.02, 0.062, 0.19, 0.56

SPR analyses were performed with the recombinant purified Hck SH3 domains covalently attached to the biosensor chip, as described under Materials and Methods. Each untagged, purified Nef protein analyte was serially injected and the Nef-Hck interaction monitored over the range of Nef concentrations shown (SF2 and Consensus variants, as indicated). The kinetics and affinities of binding were determined by fitting the data to a 1:1 Langmuir binding model, with the dissociation constants (K_D_) and error analysis calculated through the BIAevaluation 4.1 software suite.

*Consensus Nef is based on a sequence alignment of multiple B-clade Nef proteins [40].

## Results and discussion

### Expression and characterization of a mouse Hck gatekeeper mutant

**Figure 1:**
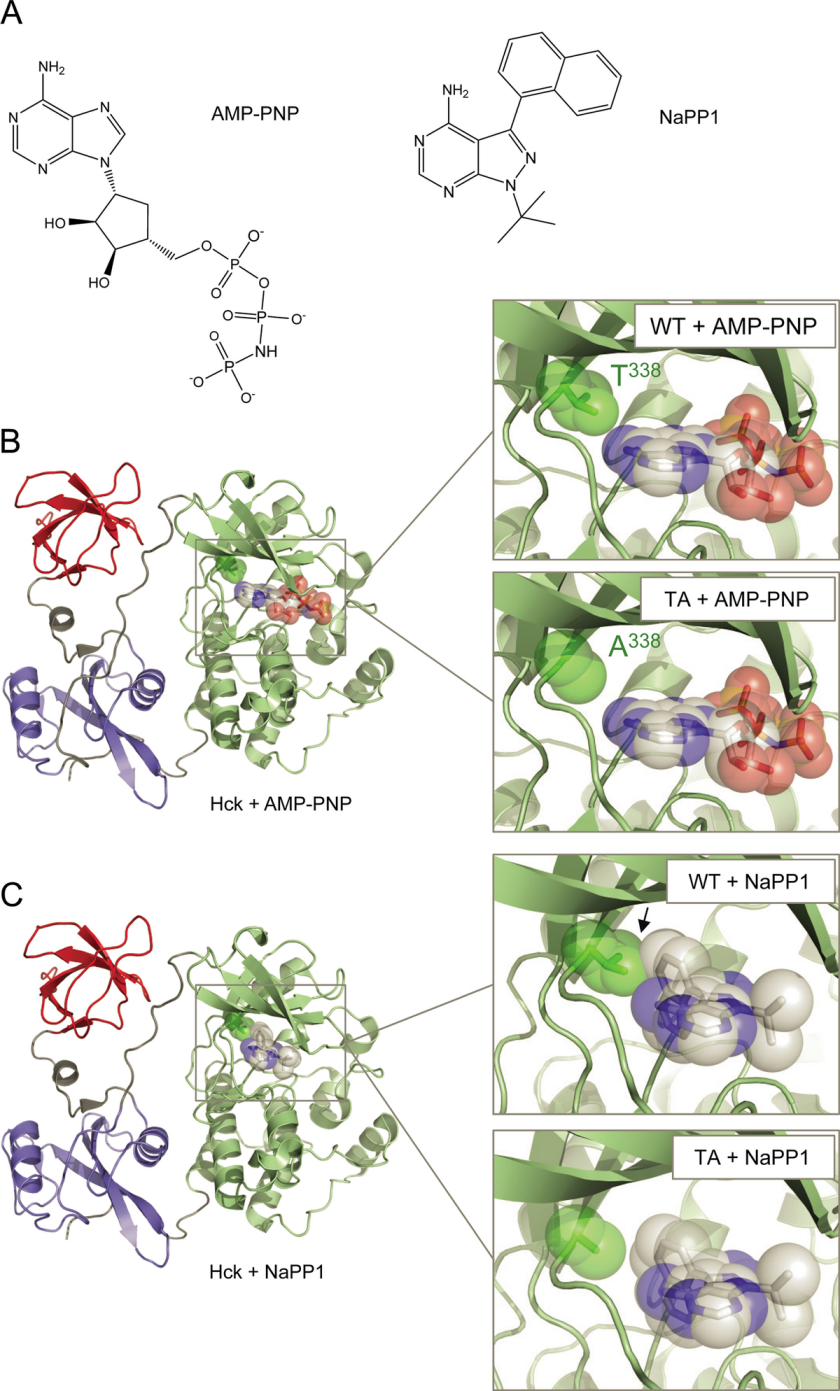
**Models of the wild-type and gatekeeper mutants of Hck in the presence of the ATP analog, AMP-PNP, and the inhibitor, NaPP1**. A) Chemical structures of AMP-PNP and NaPP1. B) Molecular model of downregulated Hck bound to AMP-PNP. The overall structure (left) shows the SH3 domain (red), the SH2 domain (blue), and the kinase domain (green). The active site is boxed, and enlarged on the right. The upper right view shows the juxtaposition of the wild-type (WT) gatekeeper residue (T338) with AMP-PNP; the view on the lower right shows the gatekeeper mutant in which Thr338 is replaced with alanine (TA). This model was produced by docking the structure of Hck-YEEI (1QCF) [20] onto an earlier structure of Hck bound to AMP-PNP (1AD5) [21]. C) Model of Hck bound to NaPP1. The overall structure is shown on the left, and is color-coded as in Part B. Close-up views of the NaPP1 binding site are shown on the right. Note that NaPP1 clashes with the side chain of the gatekeeper residue in this model (arrow; upper right), but this is relieved in the TA mutant (lower right). While the gatekeeper substitution relieves one potential steric clash with the naphthyl moiety of NaPP1, other structural changes are likely to be required to accommodate inhibitor binding. For example, the side chain of the alanine residue immediately adjacent to the DFG motif (Ala403; not shown) also clashes with NaPP1, but this clash could be relieved by movement of the DFG motif to the "out" conformation associated with kinase activation. In this case, the hydrophobic cavity would be enlarged and better able to accommodate the naphthyl moiety of NaPP1 in a horizontal orientation relative to the model shown in Figure 1C. Ultimately, an X-ray crystal structure of Hck-TA with NaPP1 bound will be required to fully understand the inhibitory mechanism. This model was produced using the crystal coordinates for Hck-YEEI bound to PP1 (1QCF) [20], and docking the NaPP1 structure onto the pyrazolopyrimdine moiety of PP1.

Previous studies have shown that Nef forms a stable complex with Hck in cells, and that this interaction results in constitutive Hck activation [19]. As described in the Background section, association with Nef may influence the structure of the Hck kinase domain active site, inducing a unique conformation amenable to selective inhibitor targeting. In order to test this idea, we engineered the active site of mouse Hck (mHck) to accommodate the pyrazolopyrimidine inhibitor, NaPP1 (Figure 1A). This modification involved substitution of the gatekeeper position (Thr338) with alanine (referred to hereafter as mHck-TA), which has been proposed to provide access for NaPP1 to the hydrophobic cavity adjacent to the ATP binding site [26,41-43]. The use of kinase domain gatekeeper mutants in combination with NaPP1 has been successfully applied in several kinase systems [26,41-45], including previous work from our group with human Hck [27].

To illustrate this chemical genetic concept, we modeled the gatekeeper alanine substitution using previous X-ray crystal structures of near-full-length downregulated Hck with either the ATP analog AMP-PNP or the NaPP1 analog PP1 bound to the active site [20,21]. As shown in Figure 1B, alanine substitution of the gatekeeper position does not appear to influence kinase interaction directly with ATP. In contrast, when NaPP1 is overlaid via the corresponding pyrazolopyrimidine of PP1, the more bulky naphthyl substituent of NaPP1 clashes with Thr338 (Figure 1C). This clash is likely to be relieved by alanine substitution at this position because of the much smaller side chain. That said, additional conformational changes in this region are likely to be required to accommodate NaPP1 (see legend to Figure 1). Nevertheless, this model provides a useful framework for understanding the relationship of the gatekeeper substitution to NaPP1 sensitivity. More importantly, these mHck-TA gatekeeper mutants enabled use of NaPP1 as a specific chemical probe for this region of the kinase domain active site. This specific kinase-inhibitor pair allowed us to ask whether HIV-1 Nef binding to the SH3 domain influences the structure of the kinase domain as described below.

### Nef does not influence wild-type mHck-TA ATP binding or competitive inhibition in solution-based kinase assays

We next expressed and purified recombinant mHck-TA with a modified C-terminal tail (YEEI), which facilitates expression and purification of the kinase in the downregulated conformation [20,46]. The K_m _for ATP was then assayed in the absence or presence of a 10-fold molar excess of two allelic variants of Nef (B-clade alleles SF2 and Consensus) using an in vitro kinase assay (Z'-Lyte) and a FRET-peptide substrate [27]. As shown in Table 2, the presence of Nef did not significantly affect the K_m _for ATP under these conditions.

**Table 2:** HIV-1 Nef binding influences the Hck active site.

Condition	K_m_, ATP (μM)	IC_50_, NaPP1 (nM)
mHck alone	12.1 ± 2.0	> 1,000
mHck + Nef-SF2	14.8 ± 1.4	> 1,000

mHck-TA alone	62 ± 12.6	53.3 ± 8.2
mHck-TA + Nef-SF2	68 ± 10.7	47.4 ± 6.0
mHck-TA + Nef-Consensus	84 ± 3.5	37.6 ± 4.6

mHck-HART alone	105.2 ± 29.0	n.d.
mHck-HART + Nef-SF2	49.34 ± 5.8	n.d.

mHck-TA-HART alone	181 ± 28.7	8.5 ± 0.7
mHck-TA-HART + Nef-SF2	69 ± 17.6	2.4 ± 0.3
mHck-TA-HART + Nef-Consensus	76 ± 23.1	3.2 ± 0.2

The K_m _values for ATP and IC_50 _values for NaPP1 were determined using the Z'Lyte assay (see Materials and Methods). Four forms of purified recombinant mouse Hck (mHck) were used in these experiments: wild-type (mHck); mHck with a modified SH3 domain high affinity RT loop for Nef binding (mHck-HART); mHck with a wild-type SH3 domain and a kinase domain mutation (T338A) that accommodates the inhibitor, NaPP1 (mHck-TA); and a double mutant (mHck-TA-HART). Experiments with Nef contained the recombinant purified viral protein at a 10-fold molar excess, and were performed with two Nef B-clade variants (SF2 and Consensus) in most cases. n.d., not determined.

**Figure 2:**
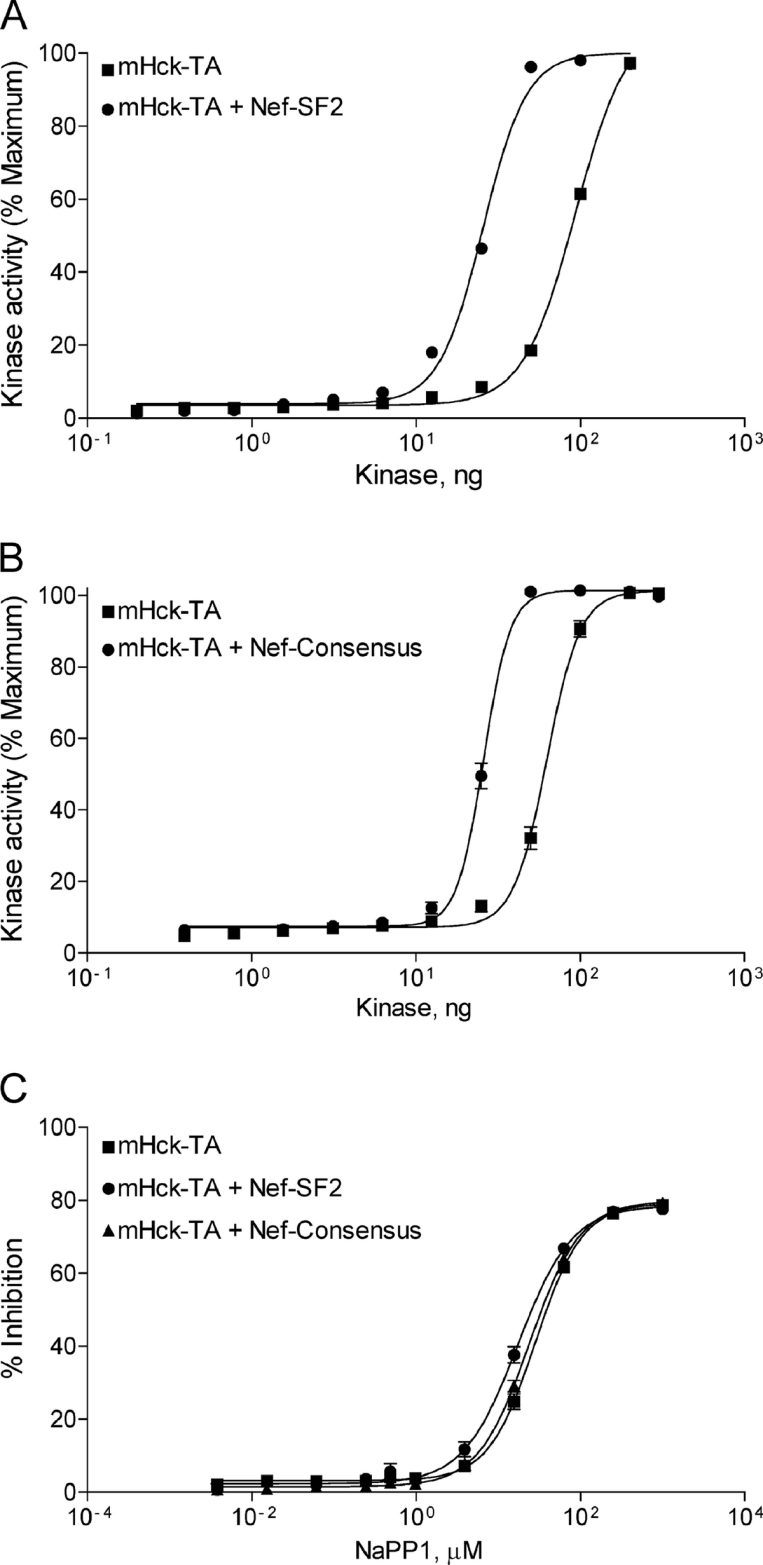
**Nef-induced activation of mHck-TA**. Recombinant downregulated mHck-TA activity was assayed over a range of kinase amounts in the presence of a 10-fold molar excess of purified recombinant Nef-SF2 (A) or Nef-Consensus (B) using the Z'Lyte method (see Materials and Methods). C) Inhibition of mHck-TA by NaPP1 in the presence or absence of Nef. Recombinant downregulated mHck-TA activity was assayed either alone or in the presence of a 10-fold molar excess of purified recombinant Nef-SF2 or Nef-Consensus over the range of NaPP1 concentrations shown using the Z'Lyte method (see Materials and Methods). All assays were performed in quadruplicate and data are shown as mean ± S.D. Each experiment was repeated twice and produced comparable results in each case.

We next performed control experiments to confirm that mHck-TA was sensitive to Nef-mediated activation in this assay. mHck-TA was assayed either alone or in the presence of a 10-fold molar excess of each Nef protein over a wide range of kinase concentrations. As shown in Figure 2A and 2B, both forms of Nef markedly shifted the mHck-TA activation curve to the left, demonstrating that Nef activates mHck in a manner quite similar to human Hck as previously reported with this assay [14,15]. Based on these results, we identified assay conditions where Hck activity was 50% of maximum in either the presence or absence of Nef. We then determined the IC_50 _value for NaPP1 while holding the ATP concentration to twice the K_m _for each condition. NaPP1 inhibited mHck-TA with equal potency in the presence or absence of Nef (Figure 2C and Table 2). Taken together, these initial observations suggested that either Nef does not influence the interaction of Hck with ATP or NaPP1, or that the proportion of Hck molecules that remain in complex with Nef was relatively low under these experimental conditions. Note that recombinant mHck without the gatekeeper substitution showed relatively low sensitivity to NaPP1 in either the presence or absence of Nef under the same conditions as mHck-TA (IC_50 _> 1000 nM; Table 2).

### mHck SH3 domain RT-loop mutants display enhanced affinity for HIV Nef

The data presented in the preceding section show that in solution, activation of mHck-TA by Nef does not perturb the K_m _for ATP or inhibitor binding. However, these assay conditions do not reflect the situation in cells, where membrane association is likely to stabilize Nef binding to Hck. Indeed, persistent interaction of Nef and Hck in cells has been recently been demonstrated using FRET [17]. In contrast, when combined in solution, the stability of the Nef:Hck complex is solely a function of their concentrations. Thus, our inability to identify changes in the K_m _for ATP or in the IC_50 _for NaPP1 following Nef-induced activation of mHck-TA may be due to dissociation of the Hck:Nef complex in solution.

**Figure 3:**
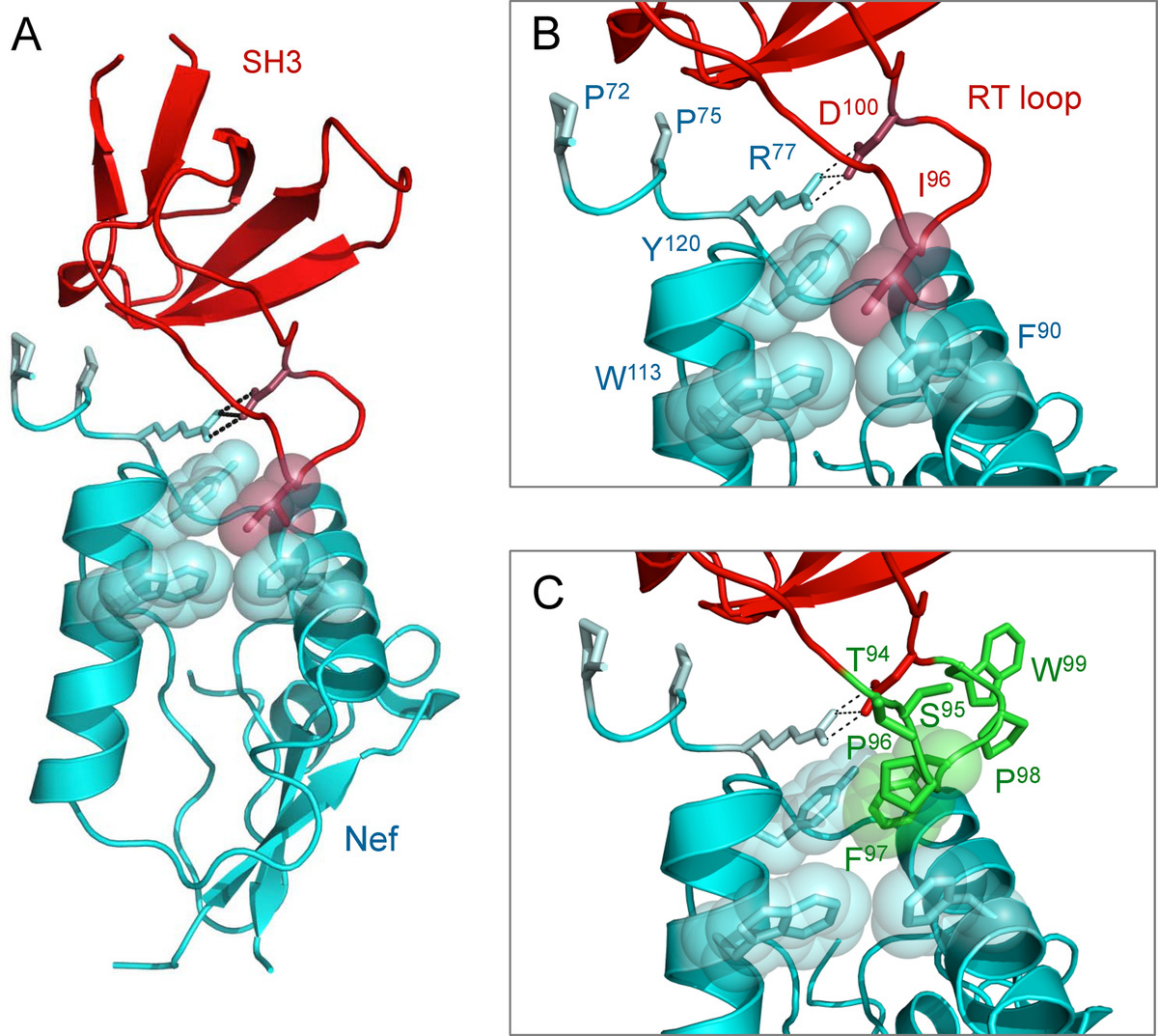
**Molecular models of HIV-1 Nef in complex with wild-type and high-affinity RT loop SH3 domains (SH3-HART)**. A) Molecular model of HIV-1 Nef (cyan) in complex with the SH3 domain of Fyn (red) in which RT loop Arg96 was replaced with isoleucine (I96) to resemble the Hck SH3 domain (PDB: 1EFN[22,49]). B) Close-up view of the wild-type interaction interface. SH3 domain RT loop Ile96 (red spheres) interacts with conserved hydrophobic residues of Nef that form a hydrophobic pocket (F90, W113, Y120). The interaction is stabilized by ion pairing of SH3 Asp100 (D100) and Nef Arg77 (R77) in the conserved PxxPxR motif (dotted lines). C) To enhance Nef interaction with SH3, the wild-type RT loop sequence E_94_AIHRE was replaced with the sequence T_94_SPFPW, which was previously reported to result in high affinity Nef-SH3 interaction [30]. The side chains of the six modified residues are modeled in green. Note that SH3-HART Phe97 (F97; green spheres) occupies the position of I96 in the wild-type crystal structure. Recent crystallographic analysis of a related high affinity Nef-SH3 interface supports this model [29].

In order to stabilize the association of Hck with Nef for solution-based assays, we modified the SH3 domain of mHck to enhance its affinity for Nef. As described under Background, one essential determinant of SH3 binding by Nef involves a conserved PxxPxR motif [22,23,47]. The affinity of the interaction between SH3 domains and their ligands is also modulated by residues in the RT-loop of the SH3 domain and areas outside of the PxxPxR motif in the ligand [30,48]. In the case of Nef, structural studies have established that residues from the Nef αA and αB helices form a hydrophobic pocket that accommodates an isoleucine residue unique to the RT loop of the Hck SH3 domain and the closely related Src-family member, Lyn (modeled in Figure 3) [22,23]. Based on these observations, Hiipakka et al. engineered enhanced SH3 affinity for Nef using a phage-display library of modified Hck SH3 domains that carry random hexapeptide substitutions in their RT-loops [30]. To increase the stability of the mHck:Nef complex in our experimental system, we selected one High Affinity RT-loop (HART) SH3 domain sequence based on its enhanced affinity for Nef as reported in this previous study [30]. In this SH3 domain, the wild-type RT-loop sequence, E_94_AIHRE, was replaced with a completely different sequence, T_94_SPFPW (Figure 3).

Before introducing the HART SH3 variant into near full-length mHck-TA, we first examined its conformational dynamics and interactions with Nef using hydrogen exchange mass spectrometry (HXMS). Wild-type mHck SH3 and the HART variant were expressed in bacteria and purified to homogeneity. Each SH3 domain was then incubated with D_2_O for various periods of time, resulting in exchange of backbone amide hydrogens in the protein with deuterium in the solvent [33,50]. Amide hydrogen bonding retards deuterium exchange and amide hydrogens with high accessibility to solvent exchange more rapidly with deuterium than those that are buried. The kinetics of exchange provides detailed information on protein fluctuations in solution. In addition, changes in the rate of protein unfolding or refolding can be correlated with the strength of ligand binding.

**Figure 4:**
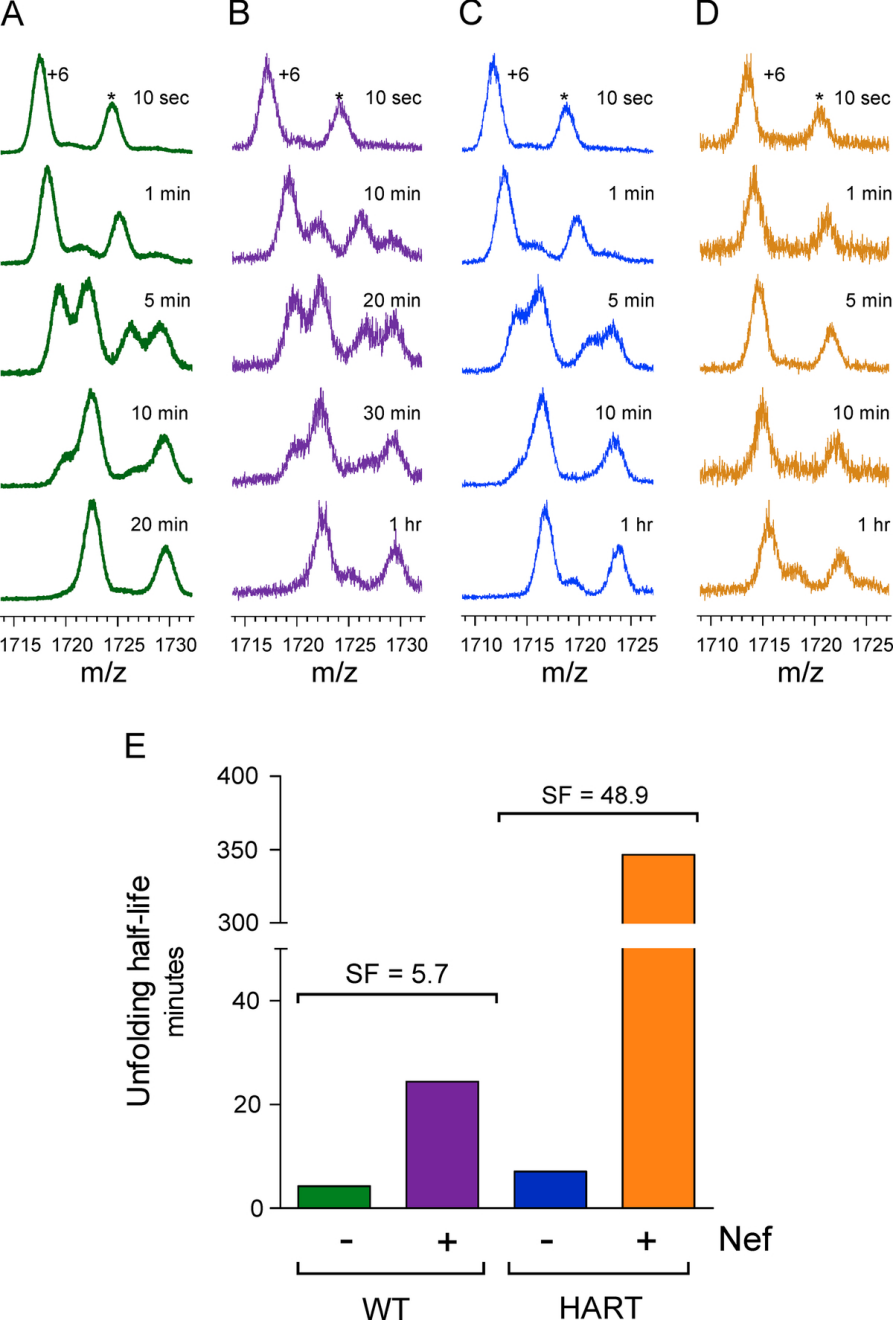
**HXMS analysis reveals that HART substitution enhances Nef:mSH3 domain interaction without influencing mSH3 dynamics**. The wild-type (WT) and HART mHck SH3 domains were exposed to deuterium in the presence or absence of Nef. Representative mass spectra showing an increase in m/z for the +6 charge state with increasing exposure time are presented for the WT mSH3 domain alone (A) and bound to Nef (B), and for mSH3-HART alone (C) and bound to Nef (D). An adduct peak (indicated by an asterisk) is also observed in each spectrum. (E) Unfolding half-lives for the SH3 domains both free and bound to Nef as well as the calculated slowdown factors (SF). The slowdown factor is a measure of the influence of Nef binding on the cooperative unfolding rate of the SH3 domain, and is calculated by taking the ratio of the unfolding half-life under different conditions as described in Hochrein, et al. [51] All HXMS studies were performed with Nef-Consensus.

Previous HXMS studies of the human Hck SH3 domain have revealed slow partial unfolding under physiological solution conditions [34]. Importantly, the dynamics of Hck SH3 domain unfolding are significantly slowed by ligand binding, particularly with Nef [34-36]. We therefore used HXMS to compare the wild-type and HART variant of the mHck SH3 domain in terms of the relative impact on cooperative unfolding, and hence binding affinity, upon full-length Nef association. As shown in Figure 4, Nef-Consensus decreased the unfolding rate of the wild-type mHck SH3 domain by approximately 5.7-fold. In contrast, Nef induced nearly a 50-fold decrease in the unfolding rate of the mSH3-HART protein. Since interaction affinity directly correlates with a proportional slowdown in the unfolding rate [19], these results demonstrate that Nef has a significantly higher affinity for mSH3-HART vs. the wild-type mSH3 domain. Note that in the absence of Nef, the half-lives of unfolding for the wild-type and HART mSH3 domains were very similar, indicating that the RT-loop modification did not influence the global dynamics of the mSH3 structure, at least within the error of determination of the half-life of the unfolding event.

**Figure 5:**
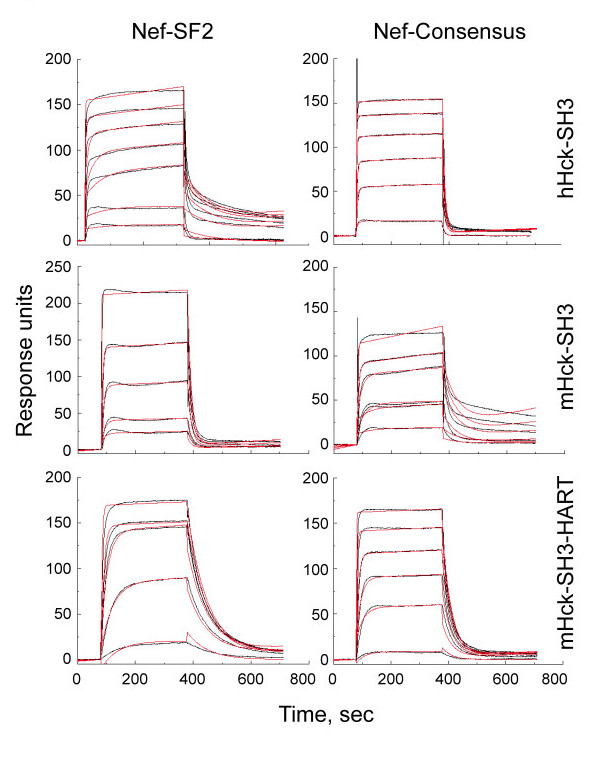
**Surface plasmon resonance (SPR) analysis of the Nef-SH3 domain interaction**. SPR analyses were performed with the recombinant purified Hck SH3 domains indicated. The SH3 proteins were covalently attached to the biosensor chip, and purified Nef proteins (SF2 and Consensus variants) were serially injected over a range of concentrations. The kinetics and affinities of binding were determined by fitting the sensorgram data (black curves) to a 1:1 Langmuir binding model (red traces) using the BIAevaluation 4.1 software suite. Nef concentrations corresponding to each response curve, along with the calculated binding constants, are presented in Table 1.

To explore the interaction of the wild-type and HART forms of the mSH3 domain with Nef in more detail, we turned to surface plasmon resonance (SPR) analysis. This approach afforded real-time determination of on- and off-rate constants as well as equilibrium dissociation constants. Representative sensorgrams are shown in Figure 5, and the resulting binding constants are summarized in Table 1. SPR analysis showed that the wild-type mouse SH3 domain bound to both Nef-SF2 and Nef-Consensus with similar affinities (0.13 vs. 0.08 μM, respectively), consistent with literature values for the human Hck SH3 domain with Nef-SF2 as determined by isothermal titration calorimetry (ITC) [29]. Introduction of the HART sequence enhanced the affinity of these interactions by 13-fold and 4-fold for Nef-SF2 and Nef-Consensus, respectively. The larger change associated with Nef-SF2 binding is reflected in an increase in the association rate constant (2.5-fold) as well as a decrease in the dissociation rate constant (nearly 5-fold). In contrast, while the association rate constant for Nef-Consensus was also enhanced with SH3-HART (6.5-fold), the dissociation rate constant was essentially unchanged (< 2-fold). The dramatic enhancement in Hck SH3-HART interaction for Nef-SF2 observed by SPR is consistent with a recent determination of Nef-SF2 interaction with a similarly modified Hck SH3 domain by ITC (10 nM via SPR vs. 12 nM via ITC) [29]. For comparative purposes, we also examined the kinetics of human Hck SH3 binding to Nef, and found that the K_D _values are similar to those observed for mouse Hck SH3 domain (Table 1). In summary, the increased affinity of SH3-HART for Nef observed by SPR is consistent with the Nef-induced decrease in the unfolding rate of SH3-HART determined by HXMS. Data from both biophysical measurements support the stabilization of Nef association with Hck for solution-based kinase assays.

### Introduction of SH3-HART sensitizes near full-length mHck to activation by Nef

**Figure 6:**
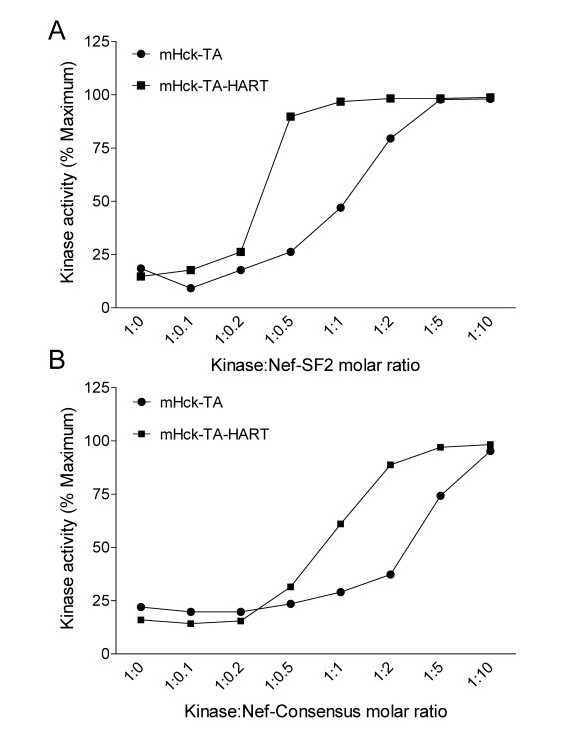
**SH3-HART substitution sensitizes mHck-TA to Nef-induced activation**. The relative kinase activities of mHck-TA and mHck-TA-HART were determined in the absence or presence Nef at each of the molar ratios shown. Experiments were performed with recombinant Nef-SF2 (A) or Nef-Consensus (B) using the Z'-Lyte kinase assay as described under Materials and Methods. All assays were performed in quadruplicate, and the data are normalized to the signal observed with a stoichiometrically phosphorylated control peptide. Results are shown as mean percent of maximum ± S.D. Both experiments were repeated twice and produced comparable results in each case.

To determine the impact of the SH3-HART modification in the context of near full-length Hck, we next introduced the HART coding sequence into mHck-TA. The resulting protein was expressed in Sf9 insect cells and purified to homogeneity. Because the HART sequence strongly enhanced interaction of the isolated SH3 domain with Nef, we predicted that mHck-TA-HART should be activated at lower concentrations of Nef than mHck-TA with a wild-type SH3 domain. To test this hypothesis, we conducted in vitro kinase assays over a range of kinase:Nef ratios for both Nef variants. As shown in Figure 6, maximal activation of mHck-TA-HART was observed at a 1 to 1 ratio with Nef-SF2, while full activation of mHck-TA required a 5-fold excess of Nef-SF2. This enhancement in sensitivity agrees with the increased affinity of Nef-SF2 for SH3-HART (Table 1). Hck-TA-HART was also more sensitive to activation by Nef-Consensus, although to a lesser extent compared to Nef-SF2. This observation fits with the smaller enhancement in SH3-HART binding to Nef-Consensus as determined by SPR.

The enhanced activation of Hck-HART by Nef most likely reflects the enhanced affinity of the modified SH3 domain for Nef. However, HART modification may also reduce SH3 affinity for the SH2-kinase linker, thus creating a partial destabilization of the downregulated conformation of Hck. To control for this possibility, we compared the basal activity of Hck-TA with and without the HART substitution in the absence of Nef. We observed that the specific activity of Hck-TA-HART was less than two-fold higher than that of Hck-TA, consistent with the idea that the enhanced sensitivity of Hck-HART to Nef is primarily due its increased affinity for Nef interaction (data not shown).

### Stable interaction with Nef impacts the mHck active site

Data presented in the previous section demonstrate that substitution of the wild-type mHck SH3 domain with mSH3-HART dramatically enhances Nef binding affinity for the SH3 domain, resulting in enhanced activation of mHck in vitro. These observations demonstrate that mHck-HART forms more stable complexes with Nef than wild-type mHck, allowing us to return to the question of whether Nef binding to the SH3 domain influences the Hck active site. We first determined whether the K_m _of mHck-TA-HART for ATP was affected by the presence of Nef. As shown in Table 2, the presence of Nef significantly reduced the K_m _for ATP with mHck-HART, and this effect was remarkably consistent for both Nef variants tested (~2.5-fold reduction in each case). A similar K_m _effect of Nef-SF2 was observed with recombinant mHck-HART in the absence of the TA mutation (Table 2). We next investigated whether the presence of Nef also affected inhibition of the Hck-TA-HART kinase by NaPP1, which occupies a binding site adjacent to and overlapping with the ATP binding site (modeled in Figure 1B). Here again, Nef-SF2 increased the sensitivity of mHck-TA-HART to inhibition by NaPP1 by more than three-fold (Table 2). Nef-Consensus also increased the apparent potency of NaPP1, albeit to a lesser extent. Together with the effect on the K_m _for ATP, these data provide strong evidence that the presence of Nef influences the conformation of the Hck active site.

## Conclusions

In the present study, we developed a modified form of mHck that enabled an assessment of the impact of Nef binding on the kinase active site under solution assay conditions. Two important modifications were involved. First, the kinase domain gatekeeper residue (Thr338) was replaced with alanine, enabling site-specific inhibition with the pyrazolopyrimidine, NaPP1. Second, the RT-loop of the SH3 domain was modified to enhance Nef binding, thus promoting stable interaction in solution as demonstrated by SPR and HXMS. Using this modified form of Hck, we showed that Nef caused a significant decrease in the K_m _for ATP as well as an increase in the apparent potency of the site-specific inhibitor, NaPP1. These findings provide evidence that Nef binding not only dislodges the SH3 domain from its regulatory position on the back of the kinase domain, but also influences the conformation of active site residues involved in ATP binding as well as inhibition by NaPP1. Because Nef interacts with and activates Hck through its SH3 domain, rather than through direct interaction with the kinase domain, our results suggest that Nef influences the conformation of active site though an allosteric mechanism. Nef-mediated activation of Hck may alter the kinetics of kinase activity as well as substrate selection. Data presented here also suggest that this novel form of Hck will enable future high-throughput chemical library screens for next-generation compounds with enhanced specificity for the Nef:kinase complex. While use of the modified form of Hck described here may simplify high-throughput screening of chemical libraries, future work must ultimately address the sensitivity of myristoylated, full-length Nef and Hck complexes to inhibitors in the context of biological membranes.

## Authors' contributions

TP-D created the modified Hck-HART coding sequences, expressed and purified the corresponding near-full-length Hck proteins, generated the kinase activity and kinetic data and helped to draft the manuscript. STS generated additional kinase and kinetic data and helped to draft the manuscript. TEW performed all HXMS studies. JJA and HS performed the SPR analyses and helped interpret the data. PN expressed and purified the recombinant HIV-1 Nef proteins for SPR analyses. JAM expressed and purified the recombinant SH3 domain proteins for SPR and HXMS analyses. JIY participated in SPR study design and data interpretation. JRE participated in HXMS study design, data interpretation, and final manuscript editing. TES participated in study design and coordination, performed the molecular modeling and wrote the final version of the manuscript. All authors read and approved the final manuscript.
